# The Impact of Prior Trial Experience on Mock Jurors’ Note Taking During Trials and Recall of Trial Evidence

**DOI:** 10.3389/fpsyg.2019.00047

**Published:** 2019-01-24

**Authors:** Joanna Lorek, Luna C. M. Centifanti, Minna Lyons, Craig Thorley

**Affiliations:** ^1^Department of Psychological Sciences, University of Liverpool, Liverpool, United Kingdom; ^2^Department of Psychology, James Cook University, Townsville, QLD, Australia

**Keywords:** juror, memory, note taking, prior experience, verdict

## Abstract

Although jurors’ recall of trial evidence is often incomplete and inaccurate, courts rely on jurors’ ability to remember trial evidence to reach just verdicts. Note taking has been found to enhance jurors’ memory of trial evidence. However, the impact of serving on multiple trials on juror note taking and recall has not been examined. Findings from the educational literature demonstrate that students who are more experienced at note taking will take more notes and recall more lecture material. Thus, the current study is the first to investigate if similar benefits are obtained in jurors. Sixty participants attended two experimental sessions and acted as mock jurors. In each session, they watched one of two trial videos, a criminal and a civil trial (order of trials was counterbalanced). All jurors were permitted to take notes whilst watching the trials. Lastly, they were asked to reach a verdict and recall as much trial evidence as they could remember (none of the jurors had access to their notes during the recall task). Jurors wrote down more correct and critical evidence during the second session when compared to the first session. However, there was no statistically significant difference between session one and session two with regards to the quantity of correct and critical evidence jurors recalled. Thus, the present study demonstrates that trial experience enhances mock jurors’ note taking, however, there is no additional enhancement regarding recall.

## Introduction

Jurors’ ability to remember trial evidence is crucial in reaching fair and informed verdicts. The evidence shows that mock jurors’ recall of trial evidence is often inaccurate (Rosenhan et al., 1994; Fitzgerald, 2000) and they tend to forget important trial information, which in turn influences their ability to reach a fair verdict (Pritchard and Keenan, 1999). Allowing jurors’ to take notes during trials has been shown to enhance their memory of trial evidence (Fitzgerald, 2000; Thorley, 2016; Thorley et al., 2016). Further, the quantity of trial evidence recorded in notes predicts how much they subsequently remember (Rosenhan et al., 1994). Although note taking has been shown to facilitate recall of trial information, all of the empirical studies assessed juror note taking and recall on a single mock trial. However, in real life jurors are sometimes required to serve on more than one trial. To our best knowledge, no study has investigated the role of serving on more than one trial has on note taking and recall. It may be that jurors’ note taking skills improve over time as they have a newly acquired understanding of trial proceedings which may enhance their note taking style. Thus, we examined the role that prior trial experience has on note taking during trials and recall of trial information. Further, we investigated whether the quantity of critical trial evidence jurors recalled was associated with their verdicts.

There are variations in practice regarding to whether jurors can take notes during a trial. For example, in England, Wales, Ireland, and some United States, jurors are guaranteed the right to take notes whereas courts in Australia, Canada, New Zealand, and other United States only permit note taking at the judge’s discretion. Studies have consistently shown that mock jurors who take notes during a trial freely recall more trial information (ForsterLee and Horowitz, 1997; Fitzgerald, 2000; Thorley et al., 2016) and provide more complete and accurate answers to cued-recall questions about a trial (Hope et al., 2014) than those who do not take notes. Therefore, the evidence suggests that note taking facilitates recollection of trial information.

The facilitative effects of note taking on recall have been explained by two theories (Di Vesta and Gray, 1972). First, note takers engage in deeper processing of the presented information during encoding, which results in an improved recall (review by Kobayashi, 2005). This is referred to as the encoding effect. However, having notes available for later restudy can also be an effective memory aid (review by Kobayashi, 2006). This is referred to as the external storage effect. Studies investigating the benefits of note taking during trials have demonstrated that having access to notes at retrieval did not result in a significant improvement in mock jurors’ recall (ForsterLee et al., 1994; Thorley et al., 2016). Thus, it could be argued that jurors benefit from note taking at encoding due to generative processing of the trial evidence. It allows individuals to store the newly learned information in a meaningful and organized way by creating links and connections between the newly acquired information (Wittrock et al., 1975; Wittrock, 1992). Storing information in an organized way aids recall as one piece of information triggers memory for related information (Tulving, 1983; Mayer, 1996).

The empirical evidence demonstrates that note taking benefits jurors. However, when real jurors were asked about note taking during trials, they indicated that they were not certain whether they should take notes (Matthews et al., 2004). Additionally, jurors had difficulty knowing what and how much to write down during a trial. Having prior experience of note taking during trials may help jurors to know what and how much to note down. Real jurors are sometimes required to serve on more than one trial. The UK government website confirms that individuals may be on a jury for more than one trial during their service (Jury service, 2018). Additionally, after 2 years of completing jury service individuals have a chance of being selected again and if they are summoned, they must serve as a juror. A United Kingdom survey found that 19% of 361 real jurors had previously experience of serving as a juror (Matthews et al., 2004). Further, an analysis of 206 United States trials revealed that 58% of the juries included jurors who had prior jury service experience of either one or two trials (Werner et al., 1985). Another study examined 175 trials and reported that 82% of the trials had at least one juror with prior experience (Dillehay and Nietzel, 1985). In addition, Dillehay and Nietzel (1985) have found that out of 902 jurors 20% served on one previous trial and 14% served on two previous trials.

To date there is no empirical research investigating the role that experience in note taking plays in juror note taking during trials. However, a limited number of studies in the educational psychology literature have explored the associations between students’ experience and note taking within lectures. First year students were found to have poor note taking skills, recording only 11% of the lecture content (Hartley and Marshall, 1974). Contrastingly, third year students were found to note down 24% of the lecture material (Hartley and Cameron, 1967), suggesting that note taking experience facilitates students’ note taking. Nye (1978) has demonstrated that first year male students’ lecture notes contained less words, and fewer main and minor points when compared to second and third year students. Others have extended this by reporting that age was positively associated with the number of important information points noted down, as well as the number of words (Wilding and Hayes, 1992). Further, older students self-reported more confidence in their note taking abilities (Carrier et al., 1988). Students, like jurors, do not receive formal instructions on how to take notes; thus it can be presumed that their note taking improves due to experience (Williams and Eggert, 2002). The findings suggest that students’ note taking skills improve with experience and over time. Although similar trends may be found with jurors, one noticeable difference between students and jurors is that students engage in note taking during lectures a lot more frequently and regularly when compared to jurors taking notes during trials. We examined whether experience plays a role in juror note taking, such that more experienced jurors are able to note down more trial information and more critical trial evidence.

Mock jurors who forget important incriminating evidence have been shown to be more likely to reach a not guilty verdict (Costabile and Klein, 2005). In our previous work (under review), we have shown that jurors who recalled greater indices of incriminating evidence were more likely to reach a guilty verdict, whereas jurors who recalled more non-incriminating evidence were less likely to find the defendant guilty. Thus, we showed that the critical trial evidence recalled was directly associated with verdicts. In the present study, we explored this association further using a civil trial video as well as the criminal trial used in our previous studies.

The main aim of the present study was to investigate the impact that serving on multiple trials has on mock jurors’ note taking. All participants attended two experimental sessions. In each session, they watched a trial video whilst taking notes. They then had their notes confiscated. After that, they reached a verdict, then freely recalled the trial, and completed a recognition task asking them about the trial.

In line with the findings from the educational psychology literature e.g., (Nye, 1978), we expected that jurors would record many more pieces of correct trial information and critical evidence in their notes during session two when compared to session one. We also predicted they would also be likely to recall a greater quantity of correct trial information and critical evidence in session two than session one. In addition, jurors would potentially score higher on the recognition task in session two as opposed to session one, as they potential note down and recall more critical information over time. If the present study finds that jurors do benefit from prior experience of note taking during trials (by noting down more trial evidence and/or recalling more trial evidence), it may be advantageous to provide real jurors with training in note taking or expose them to a mock trial in order to gain experience prior to the real trial.

We also explored whether the quantity of incriminating and non-incriminating evidence recalled during each session would influence jurors’ verdict. In our previous work (under review), we found that in a criminal trial, mock jurors who remembered the most incriminating evidence were more likely to reach a guilty verdict. Thus, we hypothesized that a similar effect would be observed in the present study. We also expected to find similar trends in the civil trial, such that jurors who remember more incriminating (or non-incriminating) evidence are more likely to reach a legally culpable (or not culpable) verdict.

## Materials and Methods

### Participants

Sixty participants acted as mock jurors (6 male participants). All were between 18 and 24 years of age (*M* = 18.8, *SD* = 1.0). Participants were drawn from a first year psychology undergraduate student sample at a northwest English university and received a payment in the form of course credit. All were eligible for jury service in England and Wales. No difference has been found regarding verdicts reached by mock jurors drawn from student versus non-student populations (Bornstein and Greene, 2011). This study was carried out in accordance with the recommendations outlined in the Declaration of Helsinki (1964). The present study was approved by the Ethical Committee of the Department of Psychological Sciences (University of Liverpool). Written informed consent was obtained from all participants.

### Design

The current study had a within-subjects design. The independent variable was time (session one and session two). The main dependent variables were: (1) the quantity of correct trial information noted down, (2) the quantity of correct trial information freely recalled; (3) the quantity of critical trial evidence noted down (critical trial evidence refers to the most important trial evidence that may influence jurors’ verdicts); (4) the quantity of critical trial evidence freely recalled; and (5) the verdict.

### Stimuli

#### Trial Videos

Two trial videos were used in the present study: a criminal trial and a civil trial. The criminal trial was a 30-min video of a 1992 murder re-trial with the case name New Jersey vs. Daniel Bias. In this trial, the defendant was accused of murdering his wife by shooting her in the head. The defendant claimed he was innocent and that his wife shot herself. The video was edited so that it contained the opening statements, the cross-examination of six witnesses and the defendant, the closing statements, and the judicial instructions. The verdict is not shown. Past research has shown non-note taking mock jurors are evenly split between guilty and not guilty verdicts for this trial (Pritchard and Keenan, 1999; Ruva et al., 2007; Hope et al., 2014).

The civil trial was a 35-min video of a civil mock case, Payne v. Davis. Davis was accused of being negligent which resulted in Payne suffering damages. Payne stopped at a red traffic light and Davis drove her car into the rear of Payne’s car. The prosecution argue that Davis was distracted and is responsible for damages that Payne suffered. The defense argue that Payne’s car began to move when the light turned green, however, suddenly she stopped. Davis slammed on the brakes to avoid the accident but she collided with Payne’s car. The trial included statements from the prosecution and defense, cross examination of four witnesses, and judicial instructions. There is no verdict, allowing participants to reach their own verdict.

#### Notepad and Free Recall Task

Consistent with real trials in England and Wales, jurors were provided with blank lined notepads and pens for note taking. Previous studies found no differences in the quantity of trial information recalled between those who were and were not allowed to access notes when recollecting the trial information (e.g., ForsterLee et al., 1994). In addition, real jurors may not always have access to their notes during deliberations (e.g., Lloyd-Bostock, 2007). Therefore, we did not include the access to notes condition in the present study. Not allowing jurors to have access to their notes at retrieval allowed us to provide a purer examination of the impact of the act of note taking during trials on recall of trial information.

All mock jurors were also given a demographic/verdict questionnaire asking them their age, gender, and whether they considered the defendant to be guilty/not guilty (criminal trial) or legally culpable/not culpable (civil trial). Finally, a 10-page A4 lined booklet was provided for the free recall test.

#### Recognition Tasks

There were two recognition tasks, one for each of the trial videos. The criminal trial task consisted of 24 true-false statements about the trial evidence. The civil trial task consisted of 20 true-false statements about the trial evidence. In each task, half of the statements were true. For counterbalancing purposes there were two versions of each task. True statements in one version were turned into false statements in the other. For instance: a true statement from the civil trial stating “Davis was using a handheld mobile phone at the time of the incident” was changed to a false statement stating “Davis was using a hands-free mobile phone at the time of the incident.” One point was awarded for each correct answer and converted into percentages of correct answers.

### Procedure

Participants attended two experimental sessions, 1 week apart. They were tested in pairs. Each participant was seated at an individual PC. During each session they watched one of the two trial videos. The order of the videos was counterbalanced (half of the participants saw the criminal trial in session one and the civil trial in session two, whereas the other half of the participants saw the civil trial in session one and the criminal trial in session two). Jurors were informed that they would be allowed to take notes during the trial and were provided with a notepad and pen. Immediately after each trial, participants had their notes confiscated. They then completed the demographic questionnaire/verdict questionnaire. Next, they completed a free recall task with no time limit. Participants were instructed to write down all trial information they could remember. Then, all participants were given the recognition test with no time limit. Lastly, participants were asked to complete the Triarchic Psychopathy Measure (Patrick, unpublished). This was included in the study as a separate investigation exploring the effects of psychopathic traits on verdicts. The analysis of this data does not appear in the manuscript, so as not to distract from the main research questions. All jurors were debriefed and the study ended.

### Coding of Notes and Recall

All notes and free recall responses were scored for the quantity of correct information. We used coding schemes which were based on a trial transcript and contained all trial information that appeared in each video. There were 207 pieces of evidence in the criminal trial and 417 pieces of evidence in the civil trial. A correct piece of trial information was given a single point if it appeared in the trial and was correctly noted down/recalled. Some information is repeated in the videos and participants often write down the correct information but fail to specify the source of this information. To ensure equivalence in the scoring across conditions, any repeated trial information was scored only once in the note taking and free recall results regardless of how many times a participant wrote this information down. Each correct piece of information (something that appeared in the trial and was correctly described) was awarded a single point. The tallied points provided two separate scores for each participant: the quantity of correct information noted down and quantity of correct pieces of trial information recalled. There were very few instances of trial information being incorrectly noted down (*M* = 0.05, *SD* = 0.20) and recalled (*M* = 0.11, *SD* = 0.31). Therefore, we excluded this from our analysis.

Further, notes and free recall responses were scored for the quantity of the critical trial evidence. Two pilot studies were conducted to establish the most important evidence that could influence a juror’s verdict in each trial. In the pilot studies, different groups of participants watched the two trial videos. They were then asked to write down the ten most important pieces of trial evidence they believe could impact upon jurors’ verdicts, and to indicate whether each piece of evidence implied that the defendant was guilty or not guilty. Next, they ranked these pieces of evidence from the most important to the least important. Sixteen unique pieces of important trial evidence were identified in the criminal trial, and 12 pieces of evidence were identified in the civil trial. Half of these implied that the defendant was guilty (henceforth called Incriminating Evidence) whereas the other half implied that he was not guilty (henceforth called Non-incriminating Evidence). For instance, the fact that the victim was right handed but was shot on left hand side of her head implies the defendant was guilty, so is an example of incriminating evidence in the criminal trial. Conversely, the fact that the victim had previously threatened to kill herself implies that the defendant was not guilty, so is an example of non-incriminating evidence in the criminal trial. In the present study, the tally of the points for critical evidence (based on the pilot studies) provided two separate scores: the quantity of incriminating evidence and the quantity of non-incriminating evidence.

Three raters blind to the experimental aims scored one-third of notes and free recall responses. The inter-rater agreement was 93% for notes and 95% for free recall responses. All disagreements were resolved by the lead author and an independent reviewer who compared the scoring and determined the correct scoring.

## Results

### Correct Trial Information

**FIGURE 1:**
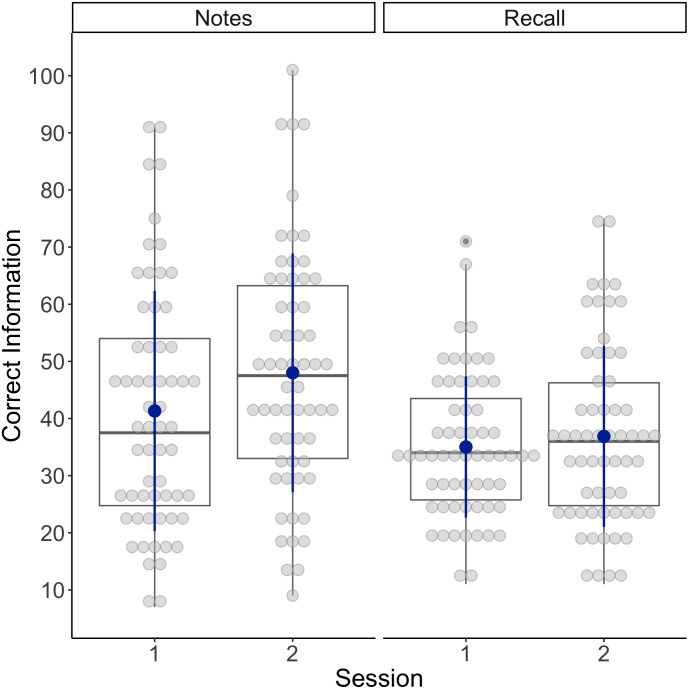
Box plots showing the individual scores (gray dots) and the means (blue dots) for the number of correct pieces of information noted down/recalled during session one and session two.

A paired samples *t*-test was conducted to investigate whether there was a significant difference between the quantity of correct trial information noted down during session one and session two. Jurors noted down more correct trial information during session two (*M* = 48.00, *SD* = 20.90) than session one (*M* = 41.32, *SD* = 21.09), *t*(59) = 2.40, *p* = 0.01, *d* = 0.31 (see Figure 1.). We also examined whether there was a significant difference between the quantity of correct trial information recalled from session one and session two. The paired samples *t*-test revealed no significant differences between the quantity of correct information jurors recalled in session one (*M* = 35.02, *SD* = 12.37) when compared to session two (*M* = 36.87, *SD* = 15.83), *t*(59) = 0.87, *p* = 0.195, *d* = 0.11 (see Figure 1). We conducted a further analysis using percentages of correct information noted down/recalled as the dependent variable in order to take into account the uneven numbers of total pieces of information included in each trial video (207 in the criminal trial and 417 in the civil trial). The percentage correct variable was computed by dividing the number of correct pieces of trial information by the total amount of information included in the trial video and converting it into percentages. This analysis confirmed the results presented here (see Appendix A for the results).

We also examined whether there were any differences in the proportion of trial information noted down and recalled (percentage) between the two trial videos. A paired samples *t*-test revealed that jurors noted down proportionally more correct trial information during the criminal trial (*M* = 18.11%, *SD* = 8.93) than the civil trial (*M* = 12.43%, *SD* = 5.13), *t*(59) = 6.32, *p* < 0.001, *d* = 0.82. They also recalled proportionally more correct trial information during the criminal trial (*M* = 14.16%, *SD* = 5.38) than the civil trial (*M* = 10.21%, *SD* = 3.32), *t*(59) = 8.01, *p* < 0.001, *d* = 1.03.

### Critical Trial Evidence

**FIGURE 2:**
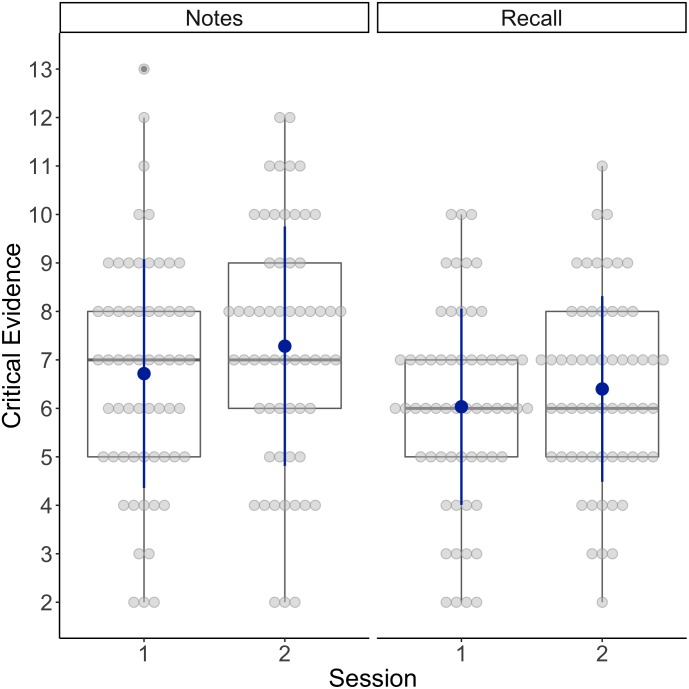
Box plots showing the individual scores (gray dots) and the means (blue dots) for the number of critical pieces of evidence jurors noted down and recalled in session one and session two.

We also conducted a paired samples *t*-test to assess whether there was a significant difference between the quantity of critical trial information noted down during session one and session two. The difference was statistically significant, *t*(59) = 1.99, *p* = 0.03, *d* = 0.26, with jurors noting down more critical trial evidence during session two (*M* = 7.28, *SD* = 2.47) than session one (*M* = 6.72, *SD* = 2.36) (see Figure 2). For the quantity of critical evidence recalled, a paired samples *t*-test showed no significant difference between the quantity of critical evidence recalled during session one (*M* = 6.03, *SD* = 2.03) and session two (*M* = 6.40, *SD* = 1.92), *t*(59) = 1.31, *p* = 0.10, *d* = 0.17 (see Figure 2).

### Recognition of Trial Information

Lastly, we examined whether participants were better at recognizing true trial information (percentage of correct answers) in session two as opposed to session one. A paired samples *t*-test revealed no significant differences between session one (*M* = 81.20, *SD* = 7.82) and session two (*M* = 83.3, *SD* = 9.87), *t*(59) = 1.27, *p* = 0.104, *d* = 0.24.

### Correlations Between Notes and Recall

We explored the association between the quantity of correct notes taken and correct information recalled during session one and session two (see Table 1), since noting down many more pieces of correct information should lead to better recall in both sessions, and possibly differently over time. That is, the benefits of note-taking on recall might be enhanced with experience by making note taking more effective over time.

**Table 1:** Descriptive statistics and zero order correlations between the quantity of correct trial information noted down and recalled during session one and session two.

Variable	Mean(± SD)	1	2	3
(1) Correct notes S1	41.32(± 21.02)	–		
(2) Correct recall S1	35.02(± 12.37)	0.630^∗∗^	–	
(3) Correct notes S2	48.00(± 20.90)	0.472^∗∗^	0.454^∗∗^	–
(4) Correct recall S2	36.87(± 15.83)	0.039	0.333^∗^	0.753^∗∗^


*^∗^*p* < 0.01 and ^∗∗^*p* < 0.001.*

We used the cocor R package version 1.1-3 (Diedenhofen and Musch, 2015) to compare two correlations measured on dependent groups, the correlations have no variable in common. Specifically, we used the Steiger’s (1980) test equation to compute the asymptotic covariance of the estimates. Each correlation coefficient is converted into a z-score using Fisher’s r-to-z transformation. The result is a z-score which is compared to the normal distribution. Values greater than 1.96 are considered significant if a 2-tailed test is performed. We compared the differences in the strength of two correlations: (a) the correlation between quantity of notes and recall during session one with (b) the correlation between quantity of notes and recall during session two. The test was not statistically significant, *z* = -1.34, *p* = 0.18. This indicates that there is no significant difference between the two correlation coefficients, suggesting that there was no significant improvement over time, such that note taking became more strongly associated with recall over time. In addition, we used the Zou’s (2007) test to calculate the confidence intervals of the difference between the two correlation coefficients. The test was not statistically significant, 95% CI -0.319, 0.057. This further confirms that the difference in the magnitude of the correlation is not statistically significant. This means that there was not a significant improvement with regards to the strength of the correlations between the quantity of correct notes taken and correct information recalled during session one and session two. The correlation coefficients used in this analysis are reported in Table 1.

We also examined the association between the quantity of critical evidence noted down and recalled during session one and session two (see Table 2). As in the previous example, we compared the differences in strength or magnitude between the correlation coefficients but this time between the quantity of critical evidence noted down and recalled during session one with that of session two. Steiger’s test showed that there was no significant difference between the two correlation coefficients, *z* = 0.60, *p* = 0.55. In addition, the Zou’s (2007) test was also not significant, 95% CI -0.186, 0.353. Again, this means that there was not a significant improvement over time with regards to the association between noting down critical trial evidence and later recall.

**Table 2:** Descriptive statistics and zero order correlations between the quantity of critical trial evidence noted down and recalled during session one and session two.

Variable	Mean(± SD)	1	2	3
(1) Critical notes S1	6.72(± 2.36)	–		
(2) Critical recall S1	6.03(± 2.02)	0.474^∗∗^	–	
(3) Critical notes S2	7.28(± 2.47)	0.584^∗∗^	0.422^∗∗^	–
(4) Critical recall S2	6.40(± 1.92)	0.161	0.394^∗^	0.391^∗^


*^∗^*p* < 0.01 and ^∗∗^*p* < 0.001.*

### Verdict and Order Effects

We conducted two logistic regressions to investigate whether the order of trials was related to jurors’ verdicts on each of the trials. The order in which the jurors were presented the trial videos did not predict their verdicts on the criminal trial, (χ^2^(1) = 0.28, Cox and Snell *R*^2^ = 0.005, Nagelkerke *R*^2^ = 0.006, *p* = 0.60). Further, the trial order did not predict jurors’ verdict on the civil trial, (χ^2^(1) = 2.45, Cox and Snell *R^2^* = 0.04, Nagelkerke *R^2^* = 0.05, *p* = 0.12).

### Critical Trial Evidence and Verdicts

Using two separate logistic regression analyses (statistically predicting effects in the civil trial separate from the criminal trial), we examined whether the quantity of incriminating and non-incriminating evidence statistically predicted the likelihood of participants reaching a guilty/legally culpable verdict. Table 3 shows the correlations between the independent variables in the logistic regressions. In the criminal trial 31 jurors reached a guilty verdict and 29 reached a not guilty verdict. In the civil trial 31 jurors reached a culpable verdict and 29 reached a not culpable verdict.

**Table 3:** Descriptive statistics and zero order correlations between the main variables.

Variable	Mean(± SD)	1	2	3	4	5
(1) Verdict criminal	–	–				
(2) Verdict civil	–	0.132	–			
(3) Criminal incriminating	3.13(± 1.31)	0.382^∗∗^	0.177	–		
(4) Criminal non-incriminating	2.83(± 1.55)	-0.494^∗∗^	0.004	0.211	–	
(5) Civil incriminating	2.60(± 1.11)	-0.17	0.316^∗^	0.271^∗^	0.364^∗∗^	–
(6) Civil non-incriminating	3.93(± 1.23)	-0.053	0.217	0.163	0.206	-0.007


*^∗^*p* < 0.05 and ^∗∗^*p* < 0.01.*

In the regression for the criminal trial, we assessed whether the quantity of incriminating evidence and the quantity of non-incriminating evidence jurors recalled predicted their verdict (0 = not guilty, 1 = guilty). The overall model significantly predicted the likelihood of jurors reaching a guilty verdict, correctly identifying 83.3% of cases (χ^2^(2) = 39.70, Cox and Snell *R*^2^ = 0.48, Nagelkerke *R*^2^ = 0.65, *p* < 0.001). The quantity of incriminating evidence recalled statistically and positively predicted the likelihood of guilty verdicts being reached by jurors, *B* = 1.68 (*SE* = 0.48), Wald = 12.01, *p* < 0.001; OR = 5.35, 95% CI = 2.07, 13.82, such that for every additional piece of incriminating evidence recalled, jurors were 5.35 times more likely to reach a guilty verdict. Further, the quantity of non-incriminating evidence recalled negatively predicted the likelihood of guilty verdicts, *B* = -1.50 (*SE* = 0.40), Wald = 14.35, *p* < 0.001; OR = 0.22, 95% CI = 0.10, 0.49, such that for every piece of non-incriminating evidence recalled, jurors were 4.55 times less likely to reach a guilty verdict.

The second logistic regression assessed whether the quantity of incriminating and non-incriminating evidence jurors recalled predicted their verdicts (0 = not legally culpable, 1 = legally culpable) on the civil trial. The overall model significantly predicted the likelihood of jurors finding the accused culpable, correctly identifying 71.7% of cases (χ^2^(2) = 8.19, Cox and Snell *R^2^* = 0.13, Nagelkerke *R^2^* = 0.17, *p* = 0.02). The quantity of incriminating evidence recalled statistically predicted jurors finding the accused culpable, *B* = 0.68 (*SE* = 0.29), Wald = 5.45, *p* = 0.02; OR = 1.98, 95% CI = 1.12, 3.51, such that for every additional piece of incriminating evidence recalled, jurors were 1.98 times more likely to reach a legally culpable verdict. However, the quantity of non-incriminating evidence recalled did not significantly predict jurors indicating a culpable verdict, *B* = -0.32 (*SE* = 0.24), Wald = 1.79, *p* = 0.18; OR = 0.73, 95% CI = 0.46, 1.16.

## Discussion

The present study examined the effect that serving on multiple trials has on juror note taking and recall of trial evidence. It also assessed whether the quantity of critical evidence jurors recall predicts their verdicts. The study found that (1) jurors wrote down more correct trial information and critical trial evidence in session two when compared to session one; (2) there was no difference in the quantity of correct trial information and critical trial evidence jurors recalled across the two sessions; (3) in both sessions there was a positive association between notes and recall with regards to correct trial evidence and critical evidence. In addition, we found that in both trials jurors who recalled the most critical incriminating evidence were more likely to find the defendant guilty/culpable. However, only in the criminal trial jurors who recalled the most critical non-incriminating evidence were more likely to find the defendant not guilty.

The present study is the first to show that jurors’ note taking improves with experience. This is in line with the findings from the educational psychology literature which suggest that students’ note taking skills improve with experience (Williams and Eggert, 2002). We found that jurors noted down not only more correct trial information but, more importantly, more critical trial evidence. Therefore, our findings demonstrate that having limited prior experience of note taking (i.e., one trial) does have a beneficial effect on jurors’ note taking during subsequent trials. Previous research demonstrates that real jurors find note taking challenging as they do not know what and how much to write down during a trial (Matthews et al., 2004). Thus, our finding is of importance as it shows that prior experience facilities note taking during trials.

The current study did not investigate the reasons why prior experience is beneficial. However, findings from the educational psychology literature demonstrate that older students self-reported more confidence in their note taking abilities (Carrier et al., 1988) and older students are better note takers (Nye, 1978). Perhaps due to their prior experience and newly acquired knowledge of trial proceedings, jurors become more confident. This may in turn help them note down more information during a trial, including more critical trial evidence. Therefore, jurors with prior experience may be more confident regarding what and how much to write down during a trial when compared to those with no prior experience. The reasons why prior experience is helpful should be investigated by future research. Although the current study shows that prior experience improves note taking, the effect sizes are relatively small which indicates that the improvements are modest. This suggests that a lack of prior experience may not have a detrimental effect on juror note taking in real trials. However, it could also be argued that jurors need more extensive prior experience in order to see greater note taking gains. This should be considered by future research.

Although note taking was found to significantly improve, there were no significant differences in the quantity of correct trial information and critical trial evidence jurors recalled across the two sessions. As the quantity of notes taken during each session was strongly associated with the quantity of recalled information, we expected recall to improve in session two when compared to session one. That is, jurors noting down more information possibly leads to a stronger association between notes and recall over time. However, the strength of the correlations between notes and recall for each session was not found to be significantly different. Therefore, the benefits of prior experience appear to be limited such that jurors note down a little more trial information after they gain experience, but they do not remember more trial information as a result of this.

There are a number of potential explanations for the non-significant difference in recall, before and after gaining note taking experience. The most straightforward explanation for not observing an improvement may be due to a small increase in the number of additional notes that jurors took in session two when compared to session one (as indicated by the small effect sizes). Perhaps jurors need to note down a larger quantity of notes in order to have a significant facilitative effect on the quantity of trial information they then recall. Alternatively, it may be that the quantity of information jurors recalled was constrained by their memory capacity. The evidence suggests that that the ability to retrieve information from long term memory may be constrained by working memory capacity (Unsworth and Engle, 2007). Perhaps jurors were able to take more notes during the second session due to prior experience, however, their working memory capacity allowed them to recall approximately the same quantity of information as they did in session one.

Furthermore, as real jurors may be permitted to access their notes when deliberating and reaching verdicts, it is important that their notes contain a large quantity of trial information, particularly the critical evidence. The present study shows that prior experience of note taking during a single trial moderately increases the quantity of correct and critical evidence jurors note down. Jurors do not appear to benefit from such small note taking enhancements when their memory is tested at an individual level. However, having more notes available during deliberations may aid real jurors’ collaborative memory and result in more informed verdicts. This should be explored by future research.

In addition, we found that verdicts for both trials were associated with the quantity of critical incriminating and non-incriminating trial evidence jurors recalled. Such that, jurors who recalled more incriminating evidence were more likely to find the defendant guilty/culpable for both trials. The present findings are in line with our previous findings (under review). We have also extended our previous findings by demonstrating that similar effects are found using a different type of trial. Therefore, our findings can be generalized to different types of trials. Further, jurors who recalled more non-incriminating evidence were less likely to find the defendant guilty but only for the criminal trial. Surprisingly, this association was not found in the civil trial. It may be that the non-incriminating evidence is weaker than the incriminating evidence in the civil trial and thus, jurors tend to remember more incriminating evidence. Given that each trial is unique in the type and quantity of evidence presented, different trials may produce slightly different effects with regards to verdicts. Future studies should investigate this with other criminal and civil trials.

### Limitations and Future Directions

One limitation is that the trials lasted only 30/35 minutes. However, as real trials can last days or weeks, this poses a threat to the ecological validity of the current findings. Note taking over longer periods of time may impose more cognitive and physical demands on jurors and impact their note taking. Future studies should explore note taking behaviors over longer periods of time. Nevertheless, we did find note taking to improve over time; such enhancement may be greater in real and longer trials. Further, the limitations of jury studies, such as the laboratory research setting and the student sample affect the current findings (Bornstein, 1999). In an attempt to reduce the impact of the laboratory environment, we used a trial video rather than a brief trial transcript (as used by others). It has been argued that using trial videos improves the ecological validity of laboratory-based juror studies (Studebaker et al., 2002). Further, our participants were undergraduate students. However, we ensured that they were eligible for jury service in the United Kingdom. Of importance, evidence suggests there is no difference with regards to verdicts reached between student and non-student samples in mock jury research (Bornstein and Greene, 2011). Lastly, the current study did not assess the impact of note taking on deliberation, and the effect of deliberation on recall. Although it is important to assess prior experience at an individual level, future research could consider investigating the role of deliberations.

## Conclusion

The present findings indicated that juror note taking improved over time. However, there was no additional enhancement regarding recall. The present study has an important applied value, such that it is the first study to demonstrate the impact of serving on multiple trials has on juror note taking. We showed that even a finite amount of prior experience is beneficial to juror note taking. However, it may be argued that the benefits of prior experience were limited as jurors noted down only a small additional amount of information during the second trial. More importantly, prior experience had no impact on the quantity of trial evidence they were able to recall. Therefore, inexperienced jurors in real trials are likely to perform as well as experienced jurors. In addition, in both trials the quantity of critical incriminating evidence jurors recall was associated with jurors being more likely to reach a guilty/ culpable verdict. This replicates our previous findings and confirms the role that critical evidence plays in verdicts.

## Data Availability Statement

The dataset for this study can be found in the OSF repository https://osf.io/jz72p/?view_only=6e74a412a0af4ff3ad49837f02a0eb88.

## Author Contributions

JL designed the study, analyzed the data, and led the writing of the article. With the supervision of CT and LC who helped design the study and aided in writing the article. ML provided critical feedback and proofread the article.

## Conflict of Interest Statement

The authors declare that the research was conducted in the absence of any commercial or financial relationships that could be construed as a potential conflict of interest.

## Appendix A: Supplementary Analysis

Two paired samples *t*-tests were conducted to assess whether jurors noted down/recalled proportionally more trial information (percentage of correct information) in session two when compared to session one. Jurors noted down proportionally more correct trial information during session two (*M* = 16.43%, *SD* = 7.85) than session one (*M* = 13.00%, *SD* = 8.34), *t*(59) = 2.82, *p* = 0.01, *d* = 0.36. However, there was a non-significant difference between the percentage of correct information jurors recalled in session one (*M* = 12.43%, *SD* = 5.51) when compared to session two (*M* = 12.17%, *SD* = 4.59), *t*(59) = 0.35, *p* = 0.729, *d* = 0.05. This analysis demonstrates that jurors did note down more during session two, however, they did not recall significantly more trial information during session two. This is in line with the findings from the main analysis examining the quantity of correct information.
